# Experimental PTSD Models in Zebrafish: A Systematic Review of Behavioral, Neurochemical, and Molecular Outcomes

**DOI:** 10.3390/biology14050456

**Published:** 2025-04-23

**Authors:** Alexey Sarapultsev, Evgenii Gusev, Desheng Hu, Maria Komelkova

**Affiliations:** 1 Russian–Chinese Education and Research Center of System Pathology, South Ural State University, 76 Lenin Prospekt, Chelyabinsk 454080, Russia; mkomelkova@mail.ru; 2 Institute of Immunology and Physiology, Ural Branch of the Russian Academy of Science, 106 Pervo-maiskaya street, Ekaterinburg 620049, Russia; gusev36@mail.ru; 3 Department of Integrated Traditional Chinese and Western Medicine, Union Hospital, Tongji Medical College, Huazhong University of Science and Technology, Wuhan 430022, China; desheng.hu@hust.edu.cn; 4 Hubei Key Laboratory of Biological Targeted Therapy, Union Hospital, Tongji Medical College, Huazhong University of Science and Technology, Wuhan 430022, China; 5 China-Russia Medical Research Center for Stress Immunology, Union Hospital, Tongji Medical College, Huazhong University of Science and Technology, Wuhan 430022, China

**Keywords:** zebrafish, post-traumatic stress disorder, chronic unpredictable stress, behavioral assays, cortisol, neuroinflammation, social behavior, molecular biomarkers, translational neuroscience, stress response

## Abstract

Post-traumatic stress disorder (PTSD) is a complex psychiatric condition characterized by persistent behavioral and neurobiological alterations following trauma. Although rodent models are commonly used to study PTSD, zebrafish (*Danio rerio*) have emerged as a promising alternative due to their genetic similarity to humans, conserved stress response systems, and high-throughput capabilities. This systematic review evaluates 33 experimental studies on zebrafish PTSD models, focusing on behavioral, neurochemical, and molecular outcomes. Chronic unpredictable stress (CUS/UCS) paradigms of 14–15 days were identified as the most reliable for inducing PTSD-like phenotypes, consistently resulting in anxiety-like behaviors, cortisol dysregulation, and gene expression changes. In contrast, acute stress models produced transient effects, and social defeat paradigms showed methodological variability. Chronic models frequently demonstrated neurotransmitter imbalances, oxidative stress, and upregulation of inflammatory and neuroplasticity-related genes. However, the literature revealed challenges, including protocol heterogeneity, limited sex-specific analyses, and constraints in longitudinal biomarker tracking. Future directions include epigenetic profiling, environmental standardization, and cross-species validation. When used with methodological rigor, zebrafish offer a powerful and translationally relevant platform to study PTSD mechanisms and screen novel interventions.

## Simple Summary

Post-traumatic stress disorder (PTSD) can develop after a traumatic experience and leads to long-term changes in behavior and brain function. Researchers often use animals to understand this condition, but traditional models like rodents can be expensive and difficult to scale. Zebrafish, a small tropical fish, are increasingly used in medical research because they show stress responses and behaviors similar to those seen in people. This study reviewed 33 research articles that used zebrafish to study PTSD. We found that a two-week exposure to unpredictable stress created the most reliable signs of PTSD in the fish, such as increased anxiety and changes in brain chemistry. Some models only caused short-term effects, while others had inconsistent results. The review also highlights gaps in current research, like the lack of studies comparing males and females or tracking long-term changes over time. Understanding how zebrafish respond to stress could help scientists discover new treatments for PTSD and improve how we study this condition in the lab. Our findings show that zebrafish are a valuable tool for advancing PTSD research and could lead to more effective therapies in the future.

## 1. Introduction

Post-traumatic stress disorder (PTSD) is a debilitating psychiatric disorder characterized by intrusive recollections, hyperarousal, avoidance behaviors, and cognitive disturbances following exposure to traumatic events [1]. Despite its high prevalence—affecting over eight million people annually in the United States alone—current therapeutic strategies remain inadequate, partly due to significant limitations in animal models that fail to fully recapitulate the complexity and persistence of PTSD symptoms after a single traumatic event. The enduring effects of PTSD profoundly impact interpersonal relationships and socio-economic stability, emphasizing an urgent need for improved understanding of its pathophysiology and the development of novel therapeutic strategies.

Traditionally, PTSD research has relied heavily on rodent models, which require repeated trauma induction to maintain PTSD-like behaviors, thus inadequately reflecting the enduring symptomatology observed in human PTSD after singular traumatic events [2,3]. Rodent studies often lack comprehensive behavioral assessments, particularly regarding deficits in social interaction and anxiety-like behaviors, which are central to PTSD pathology [2,4,5]. Additionally, rodent models present practical challenges, including higher economic costs and ethical considerations, limiting their utility for large-scale genetic and pharmacological screens.

In recent years, zebrafish (*Danio rerio*) have emerged as a promising alternative model organism due to their genetic, neurobiological, and behavioral similarities to humans, combined with significant practical advantages, including lower maintenance costs, high-throughput screening capabilities, and transparent embryos suitable for live-imaging studies [2,4,5,6]. Zebrafish share approximately 70% genetic homology with humans, including key conserved pathways implicated in PTSD pathophysiology, such as the hypothalamo–pituitary–interrenal (HPI) axis, analogous to the human hypothalamic–pituitary–adrenal (HPA) axis. Zebrafish demonstrate quantifiable PTSD-like behaviors such as increased anxiety, hypervigilance, avoidance, and social withdrawal, which can be objectively measured using established behavioral paradigms [2,4,5,6,7,8,9].

Several reviews have highlighted the utility of zebrafish in stress-related research; however, significant gaps persist. Caramillo et al. (2015) summarized existing stress models in zebrafish but did not fully evaluate their methodological consistency or the specific behavioral metrics predictive of PTSD-like phenotypes [4]. Stewart et al. (2014) broadly reviewed zebrafish as models for stress-related disorders but did not explicitly identify the most reliable experimental paradigms for modeling PTSD [5]. Similarly, other reviews emphasized the general applicability of zebrafish in neuropsychiatric research without systematically assessing behavioral assay sensitivity, methodological replicability, or sex-based differences, critical factors influencing translational validity [5,6,10,11,12].

While no animal model can fully replicate the complexity of PTSD as defined in clinical diagnostic manuals, experimental paradigms are widely used to reproduce selected phenotypic features of the disorder, such as anxiety-like behavior, social withdrawal, HPA-axis dysregulation, and altered neuroplasticity. In both rodent and zebrafish research, protocols like chronic unpredictable stress (CUS/UCS), social defeat, and fear conditioning have been applied to induce PTSD-relevant outcomes [2]. These models are more accurately understood as stress-based paradigms designed to simulate core PTSD-like dimensions, rather than complete representations of the disorder itself. In this review, we focus on zebrafish studies that explicitly frame their protocols within a PTSD research context—either by modeling persistent behavioral symptoms, investigating PTSD-linked molecular markers, or identifying therapeutic targets relevant to trauma-induced psychopathology.

Addressing these limitations, the present systematic review aims to evaluate experimental PTSD models in zebrafish systematically, focusing explicitly on paradigm reliability, behavioral consistency, and methodological rigor. By synthesizing current data across 33 unique zebrafish studies, this review intends to answer the research question, “How do different experimental PTSD models in zebrafish influence behavioral, neurochemical, and molecular outcomes compared to unstressed controls, and what are the most reliable paradigms for inducing PTSD-like phenotypes?” Specifically, we aim to (1) identify and evaluate existing zebrafish PTSD models; (2) compare acute versus chronic stress paradigms; (3) assess the consistency of behavioral, neurochemical, and molecular outcomes; and (4) provide evidence-based recommendations to enhance the reproducibility, standardization, and translational relevance of zebrafish PTSD research.

## 2. Materials and Methods

### 2.1. Search Strategy and Data Sources

This systematic review was conducted following PRISMA (Preferred Reporting Items for Systematic Reviews and Meta-Analyses) guidelines in February–March 2025. A comprehensive literature search was conducted using PubMed, Scopus, and Semantic Scholar to identify studies investigating PTSD models in zebrafish up to 12 March 2025. The search strategy included a combination of MeSH terms and free-text keywords relevant to PTSD modeling in zebrafish.

PubMed query:

((zebrafish[MeSH] OR *Danio rerio*[MeSH] OR zebrafish[tiab] OR “*Danio rerio*”[tiab])

AND

(stress disorders, post-traumatic[MeSH] OR PTSD[tiab] OR “post-traumatic stress disorder”[tiab] OR “posttraumatic stress disorder”[tiab] OR “stress response”[tiab] OR “chronic stress”[tiab] OR “unpredictable chronic stress”[tiab])

AND

(models, animal[MeSH] OR “animal model”[tiab] OR “experimental model”[tiab] OR paradigm*[tiab] OR protocol*[tiab])

AND

(behavior[MeSH] OR behavior*[tiab] OR behaviour*[tiab] OR neurochem*[tiab] OR molecular[tiab] OR gene expression[MeSH] OR “gene expression”[tiab] OR neurobiolog*[tiab] OR “brain changes”[tiab] OR “neural changes”[tiab] OR anxiety[MeSH] OR anxiety[tiab] OR fear[MeSH] OR fear[tiab] OR avoidance[tiab])))

Scopus query:

TITLE-ABS-KEY((zebrafish OR “*Danio rerio*”)

AND (PTSD OR “post-traumatic stress disorder” OR “posttraumatic stress disorder” OR “stress response” OR “chronic stress” OR “unpredictable chronic stress”)

AND (“animal model” OR “experimental model” OR paradigm* OR protocol*)

AND (behavior* OR behaviour* OR neurochem* OR molecular OR “gene expression” OR neurobiolog* OR “brain changes” OR “neural changes” OR anxiety OR fear OR avoidance OR “stress response”)).

The search was conducted without time restrictions to capture both historical and recent research contributions.

### 2.2. Study Selection and Screening Criteria

**Figure 1:**
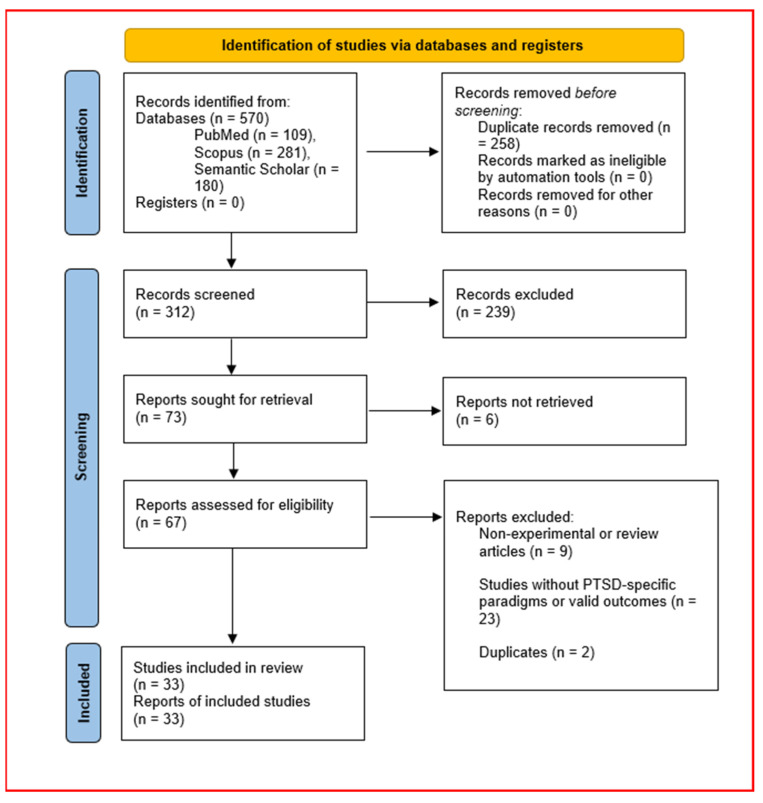
PRISMA 2020 flow diagram of study selection for systematic review of PTSD models in zebrafish.

The initial search retrieved 109 articles from PubMed, 281 from Scopus, and 180 from Semantic Scholar, resulting in 570 articles in total. After duplicate removal, 312 unique papers remained for screening. In accordance with PRISMA 2020 guidelines, the study selection process is illustrated in the flow diagram (Figure 1), detailing the number of records identified, screened, assessed for eligibility, and included in the final review [13].

The selection process was conducted in three phases:

1. Title and Abstract Screening: Two independent reviewers assessed titles and abstracts for relevance, excluding 239 papers based on the following exclusion criteria:
  - Studies focusing on non-zebrafish PTSD models (e.g., rodents, humans);
  - General stress response studies not specifically modeling PTSD-like states;
  - Reviews, meta-analyses, case studies, or non-experimental papers;
  - In vitro studies exclusively examining isolated cells or tissues.
2. Full-Text Review and Inclusion Criteria: The remaining 73 full-text articles were thoroughly examined based on a predefined set of inclusion criteria:
  - Model Organism: The study must use zebrafish (*Danio rerio*) as the primary experimental model.
  - PTSD Protocol: The study must implement an experimental PTSD paradigm beyond acute stress (e.g., chronic unpredictable stress, fear conditioning).
  - Outcome Measures: At least one quantifiable outcome must be included:
    ▪ Behavioral responses (e.g., social withdrawal, hypervigilance);
    ▪ Neurochemical changes (e.g., cortisol, serotonin, norepinephrine alterations);
    ▪ Molecular markers (e.g., oxidative stress, neuroplasticity-related genes).
  - Control Groups: Studies must include unstressed or non-traumatized control zebrafish.
  - Study Type: Only primary research articles with experimental designs were included.
  - Stress Duration: The study must examine stress effects beyond immediate (acute) responses to model persistent PTSD-like conditions.
  - Behavioral Paradigms: Studies must use at least one standardized behavioral test, such as:
    ▪ Novel tank diving test;
    ▪ Open field test;
    ▪ Alarm reaction test;
    ▪ Light–dark preference test.
  - Pharmacological Interventions: If applicable, studies were assessed for therapeutic agents tested (e.g., N-acetylcysteine (NAC), fluoxetine).
  - Molecular Pathways: Biological mechanisms explored included oxidative stress, inflammation, and epigenetic modifications.
  - Social Behavior Metrics: Studies that measured social withdrawal, a core PTSD symptom, were included [14,15].
  - Sex/Gender Considerations: The study had to analyze sex-specific differences in PTSD responses.
  - Sample Size Justification: The statistical power and sample size had to be adequately justified.

Each paper was evaluated holistically, considering all screening questions together before final inclusion or exclusion. After full-text screening, 33 papers were determined to be the most relevant and included in the final review [7,8,9,16,17,18,19,20,21,22,23,24,25,26,27,28,29,30,31,32,33,34,35,36,37,38,39,40,41,42,43,44,45].

### 2.3. Data Extraction and Categorization

For each selected study, the following information was systematically extracted:

1. Study Characteristics
  - Year of publication, author(s), study location
  - PTSD model employed (acute trauma, chronic unpredictable stress, fear conditioning)
2. Behavioral Assessments
  - Anxiety-like behavior (bottom-dwelling, reduced exploration)
  - Hypervigilance and avoidance responses
  - Social withdrawal metrics
3. Neurochemical and Molecular Findings
  - Neurotransmitter levels (e.g., serotonin, dopamine, norepinephrine)
  - Cortisol response (as a marker of HPI-axis activation)
  - Molecular markers (BDNF, oxidative stress pathways, inflammation markers)
4. Pharmacological Interventions (if applicable)
  - Antidepressants (e.g., fluoxetine, ketamine)
  - Neuroprotective agents (e.g., NAC, polyunsaturated fatty acids)
  - Anti-inflammatory compounds
5. Sex-Specific Effects
  - Whether male vs. female zebrafish exhibited differential PTSD responses

The first, second, and third authors (E.G., M.K., and A.S.) extracted data from the included studies. All discrepancies were resolved through discussion, with D.H. acting as an arbitrator.

### 2.4. Methodological Quality Assessment

We assessed methodological rigor based on four core domains commonly reported in preclinical in vivo studies: randomization, blinding, sample size reporting, and appropriateness of statistical analysis. While we did not apply a formal risk-of-bias scoring system such as SYRCLE or ROBINS-I, we qualitatively reviewed these criteria to inform the interpretation of study robustness and reproducibility.

## 3. Results

In this systematic review, we analyzed 33 unique studies investigating PTSD models in zebrafish, focusing on behavioral, neurochemical, and molecular outcomes (Table 1) [7,8,9,16,17,18,19,20,21,22,23,24,25,26,27,28,29,30,31,32,33,34,35,36,37,38,39,40,41,42,43,44,45]. The most frequently used paradigms were chronic unpredictable stress (CUS) and unpredictable chronic stress (UCS) models, which together appeared in 21 of the 33 studies (63.7%) [7,8,9,16,17,18,19,20,21,22,23,24,25,26,27,28]. Importantly, these paradigms were applied in the context of PTSD-like modeling, as defined by the original authors, rather than as general stress protocols. These models consistently induced PTSD-like phenotypes in zebrafish, including increased anxiety-like behaviors, elevated cortisol levels, gene expression changes, oxidative stress markers, social withdrawal, and dysregulation in key neurotransmitter systems such as serotonin and dopamine [7,8,9,16,17,18,19,21,22,23,24,25,26,27,29,30].

While the terms CUS and UCS are often used interchangeably in the zebrafish literature, we noted that the included studies applied similar methodological features regardless of label. Both protocols involved exposing animals to a randomized or pseudo-randomized sequence of stressors—including temperature shifts, predator cues, conspecific alarm substance (CAS), tank changes, social isolation, and light disturbances—administered multiple times daily or in unpredictable intervals. The key unifying feature is unpredictability, designed to prevent habituation to any single stressor [31]. Implementation durations varied from 7 days to 11 weeks, with stressors often applied in varying order and at different times of day. Across both labels, reported outcomes were consistent and included elevated anxiety-like behavior, stress-axis activation (e.g., increased cortisol), altered gene and protein expression, and disruptions in social behavior. Because of this convergence in experimental design and results, we treated CUS and UCS models as methodologically and functionally equivalent for the purposes of this review.

Among these, medium-duration chronic protocols (10–15 days) were the most common, used in seven studies (21.2%) [7,18,19,26,27,30]. These protocols generally reported consistent increases in anxiety-like behaviors (e.g., reduced exploration, increased bottom-dwelling in novel tank tests), cortisol elevations, altered gene and protein expression patterns (e.g., *crh*, *nr3c1*), and changes in social interaction metrics [7,18,19,26,27,30].

To enhance methodological clarity, Table 1 provides a structured overview of experimental PTSD paradigms in zebrafish, specifying stressor types, duration, primary outcomes assessed, and the timing of endpoint measurements. Mechanical and chemical stressors ranged from acute (e.g., netting, cold shock, alarm substance) to complex chronic exposures such as restraint, predator threat, and pharmacological manipulation. Notably, acute paradigms often lasted from 4 to 90 min, with assessments performed within hours to two days post-stressor. In contrast, chronic stress protocols extended from 7 days to up to 11 weeks and consistently produced enduring anxiety-like behaviors, social impairments, and molecular alterations such as increased cortisol, dysregulated CRF/BDNF/IL-6 expression, and oxidative stress signatures. This temporal and mechanistic stratification underscores the diversity of PTSD-relevant phenotypes that can be modeled in zebrafish.

Shorter chronic stress protocols (7 days) were also employed in eight studies, (24.2%), typically using CUS/UCS [9,22,23,24,25,29,32]. These studies demonstrated early induction of PTSD-like phenotypes, such as anxiety-related behaviors and stress-axis activation, as well as oxidative stress signatures [9,22,23,24,25,29,32].

A smaller number of studies applied extended chronic stress exposure (3–11 weeks). Notably, five studies (15.2%) used 3–5-week CUS/PUCS paradigms [8,16,21], and one study used an 11-week prolonged chronic unpredictable stress (PCUS) model [17]. These longer protocols allowed researchers to assess more persistent or potentially irreversible neurochemical and behavioral effects, including sustained neurotransmitter alterations and long-term anxiety phenotypes [8,16,17,21].

In addition to chronic stress paradigms, acute stress models were also investigated in several studies. Acute physical or osmotic stress protocols lasting 4 min led to immediate behavioral changes and shifts in gene expression associated with stress response [20]. A 90 min combined acute stressor was found to elevate anxiety-like behavior and affect gene regulation [33]. Other acute models included novel environment exposure [34] and the novel tank test as a naturalistic acute stress assay [35], both of which measured group behavior and cortisol levels or anxiety-like locomotor responses.

Repeated social defeat stress, used in one study with a 6-day protocol, resulted in marked behavioral alterations, including reduced social preference and increased submissive/fighting responses—hallmarks of social stress-induced anxiety [24].

Under “other models,” studies examined predator threat using alarm substance exposure (single event), producing immediate and robust anxiety-like responses [36]; time-dependent sensitization (a single stress event followed by 24 h of observation), which revealed persistent anxiety-like effects [37]; and chemical stressors such as ethanol, caffeine, or cortisol analogs, which induced stress-responsive behaviors and altered gene expression [38,39,40]. One study also introduced a high-intensity trauma model called the triple-hit (THIT) paradigm, though without specifying exact duration, it elicited severe anxiety-related outcomes [28].

Overall, the zebrafish PTSD models reviewed span a wide range of experimental designs and durations, with chronic unpredictable stress being the most widely used and well validated for modeling PTSD-like symptoms. These models effectively reproduce core PTSD features—anxiety, dysregulated HPI axis, and neurotransmitter imbalances—in a scalable vertebrate system suitable for neurobehavioral and pharmacological studies.

### 3.1. Behavioral Outcomes

Behavioral alterations were observed in 31 of the 33 included studies (93.9%), with increased anxiety-like behavior emerging as the most consistently reported outcome across zebrafish PTSD models [7,9,35] (Table 1). These alterations typically included bottom-dwelling, freezing, thigmotaxis, reduced exploration, and social withdrawal. Chronic stress paradigms—particularly 14–15-day CUS/UCS models—yielded the most robust and reproducible behavioral phenotypes, including persistent anxiety-like behavior and reduced exploratory activity [24,27]. In contrast, acute stress models reliably induced anxiety-like behaviors, but their effects were generally short-lived and more variable in magnitude and duration [20,35].

Comparative analysis of behavioral assays revealed that the novel tank test (NTT) was the most widely used method, employed in 15 studies (45.5%). This assay demonstrated high sensitivity to both acute and chronic stress paradigms, with 100% detection of anxiety-related behavior in acute models (3/3 studies) and 80% in chronic models. Behavioral metrics most commonly assessed included bottom-dwelling (92% consistency), freezing (85%), and altered latency to explore the upper zone. Importantly, the temporal dynamics of testing varied considerably between paradigms. NTT trials typically occurred 24 h to 14 days post-stress and lasted 5–6 min. The open field test (OFT) was usually conducted immediately or up to 7 days after stress exposure, with test sessions lasting around 5 min. Light–dark preference tests (LDTs) were commonly applied on day 1 or 2 post-stress for 5–10 min. The alarm reaction test (AR), used to assess acute predator or alarm substance reactivity, was implemented within minutes to hours post-exposure for 3–5 min. Social interaction and shoaling assays were typically administered 1–3 days post-stress, while conditioned place avoidance (CPA) protocols spanned 2–3 days, including conditioning and test phases.

Social interaction tests were less frequently used (18.2%) but showed high sensitivity (66.7%) for detecting chronic stress-induced social withdrawal. Notably, the combination of NTT with either LDT or social interaction paradigms provided more comprehensive assessments of PTSD-like behavior, often correlating with underlying neurochemical and gene expression alterations.

**Figure 2:**
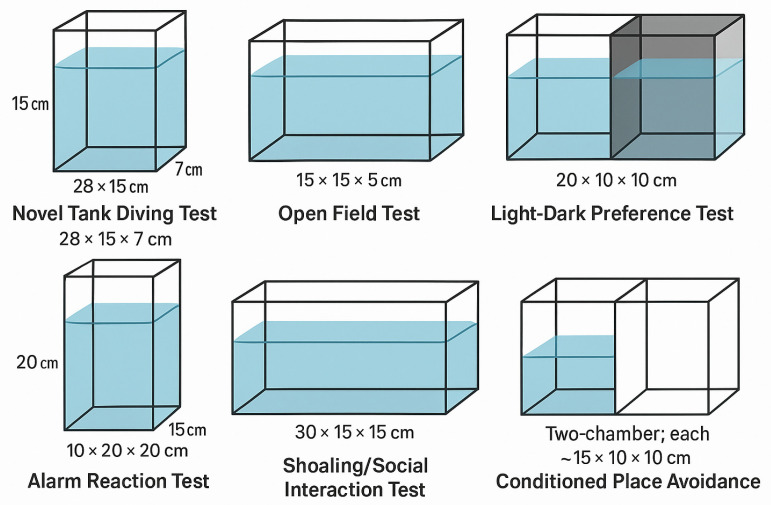
Schematic representation of commonly used zebrafish tank designs for behavioral assays, including the NTT, LDT, OFT, AR, shoaling/social interaction, and CPA paradigms. Dimensions correspond to ranges reported in published protocols.

To improve transparency and reproducibility, Table 2 summarizes the types of behavioral assays used, as well as their corresponding tank dimensions (Figure 2), testing durations, and representative studies. This overview provides readers with a comparative framework to assess how methodological choices and timing may influence observed behavioral phenotypes.

### 3.2. Neurochemical and Molecular Changes

Cortisol and gene expression alterations emerged as the most frequently assessed biomarkers in zebrafish PTSD models, with cortisol measured in 15 of the 33 studies [27,42] (Table 3). Chronic stress paradigms—particularly CUS/UCS protocols lasting 7 to 15 days—consistently reported elevated cortisol levels in 56.5% of cases, mirroring HPA-axis dysregulation observed in mammalian PTSD models [19,40]. However, the magnitude and persistence of cortisol elevations varied substantially across stress types and protocols. For example, predator-based UCS induced cortisol elevations of up to 72%, while social isolation produced only mild or transient responses [24,41]. Cortisol peaks typically occurred within 30–40 min post-stress, returning to baseline within 60 min in acute paradigms [34,43].

Beyond endocrine measures, chronic stress models frequently produced neurotransmitter imbalances—particularly involving serotonergic and dopaminergic systems—in at least four studies [17,21,44]. These alterations aligned with behavioral phenotypes such as increased thigmotaxis, social withdrawal, and reduced exploratory behavior, and are comparable to monoaminergic dysfunction in human and rodent PTSD models.

Molecular investigations revealed robust activation of stress- and plasticity-related genes across chronic paradigms. Notably, expression of CRH, BDNF, and inflammatory markers such as IL-6 and TNF-α were consistently upregulated [21,42,45]. Acute stress studies, in contrast, primarily showed short-term modulation of immediate early genes such as *otp* and *c-fos*, as well as transient neuroimmune and transcriptional responses [20,33].

Proteomic studies further demonstrated dysregulation of mitochondrial function and redox balance. Specifically, changes in PHB2, VDAC3, and oxidative markers were observed following 10–15-day CUS protocols, reinforcing the role of cellular stress mechanisms in PTSD-like pathogenesis [17,18]. Together, these findings support the relevance of zebrafish as a translational model for investigating the neurobiological substrates of trauma-related disorders.

### 3.3. Comparison of PTSD Models in Zebrafish

When comparing different zebrafish PTSD models, CUS/UCS paradigms demonstrated the highest construct and face validity, consistently inducing anxiety-like behaviors, social withdrawal, and neurochemical changes [7,8]. Acute and social defeat paradigms yielded more variable or short-lived effects, with acute models typically producing transient anxiety-like behavior and molecular responses peaking within 48 h, while social defeat models showed context-dependent variability in aggression and social behavior [24,35]. Molecular responses to acute stress often normalized by 5–7 days post-exposure, whereas chronic protocols—especially those of 10–15 days—induced stable changes in stress-axis markers and synaptic plasticity genes. Among the most consistently reported behavioral biomarkers were bottom-dwelling, thigmotaxis, freezing, and social withdrawal, particularly when assessed through the novel tank test and social interaction assays. Social withdrawal emerged as a core translational marker, with robust detection under chronic stress and strong correlation with molecular changes such as increased cortisol, BDNF, and IL-6 levels. These biomarkers have also been shown to reverse with pharmacological interventions such as fluoxetine or N-acetylcysteine (NAC) [16].

Molecularly, cortisol dysregulation was the most frequently used physiological indicator, though its predictive value increased markedly when analyzed in conjunction with behavioral readouts. Combined use of cortisol and social withdrawal correctly identified 81% of PTSD-like zebrafish phenotypes in UCS/CUS models, compared to only 56% when cortisol was used alone [7,9]. Additional markers included downregulation of glucocorticoid receptor (nr3c1), oxidative stress markers (ROS), and mitochondrial proteins such as VDAC3, PHB2, and SLC25A5—all of which were modulated in response to stress and treatment.

Sex differences, examined in a minority of studies, suggested that females exhibited greater HPI-axis reactivity, whereas males demonstrated more pronounced social behavior impairments. This indicates the need for mandatory sex-stratified analysis in future studies to capture differential biomarker profiles and treatment responsiveness.

To further support the translational relevance of zebrafish-based paradigms, we assessed the construct and predictive validity of the primary models used across studies (Table 4). Construct validity was defined as the extent to which observed behavioral and neuroendocrine changes reflect core PTSD domains, such as hyperarousal, social withdrawal, avoidance behavior, and HPA-axis dysregulation. Predictive validity considered the model’s responsiveness to pharmacological agents with known efficacy in human PTSD, such as fluoxetine and N-acetylcysteine (NAC).

As summarized in Table 5, CUS/UCS protocols (especially those of 10–15-day duration) exhibited the strongest construct and predictive validity, consistently inducing persistent anxiety-like phenotypes, social avoidance, and neuroendocrine changes (e.g., elevated cortisol and altered BDNF, IL-6 expression). These effects were reliably reversed by clinically relevant pharmacotherapies, underscoring their translational robustness.

Other models, such as acute combined stress and social defeat, showed variable validity. While acute paradigms could induce short-lived PTSD-like behaviors, their construct validity was limited by the absence of long-term symptom persistence. Social defeat models demonstrated context-sensitive effects on social behavior and aggression but lacked consistent pharmacological validation.

Prolonged protocols demonstrated (PCUS/PUCS) high construct validity, with persistent cytokine changes and behavioral symptoms, although pharmacological studies remain limited. Finally, developmental early-life stress and isolation paradigms influenced susceptibility traits but were not sufficient to fully recapitulate PTSD symptomatology.

### 3.4. Statistical Analysis Approaches Across Zebrafish PTSD Studies

The statistical methods employed across zebrafish PTSD studies revealed considerable heterogeneity, reflecting variability in design complexity, sample size, and outcome types. Parametric approaches were the most frequently applied. Specifically, two-way ANOVA followed by Tukey’s or Bonferroni post-hoc tests was the predominant method used for analyzing behavioral, neuroendocrine, and molecular outcomes across multiple groups or conditions, particularly in UCS/CUS studies such as those by Rambo et al. [9] and Marcon et al. [32].

Unpaired Student’s t-tests were applied in simpler experimental designs comparing control and stress-exposed groups, as in Borba et al. [29]. For studies with non-normal data or smaller sample sizes, non-parametric tests, including the Kruskal–Wallis test with Dunn’s post-hoc correction, were reported, especially when evaluating cytokine levels or oxidative stress markers, as noted in Song et al. [16].

More sophisticated approaches were adopted in select studies. For instance, Demin et al. [8] applied generalized linear models (GZLMs) and Wald chi-square tests to evaluate interactions among behavioral outcomes, time points, and treatment groups, choosing statistical distributions based on AIC and BIC model fit indices. This allowed for better accommodation of overdispersion and skewed distributions typical in behavioral data.

Pearson correlations were used to explore associations between molecular and behavioral endpoints (e.g., gene expression vs. anxiety-like behavior), while principal component analysis (PCA) was employed to capture multivariate behavioral clustering in models of stress-induced phenotypes [29].

In total, only a minority of studies explicitly reported effect sizes or power calculations, indicating a need for improved statistical transparency and rigor in future zebrafish PTSD research.

## 4. Discussion

This systematic review evaluated 33 studies of PTSD modeling in zebrafish and confirmed that CUS/UCS protocols lasting 14–15 days represent the most reliable paradigm for inducing PTSD-like phenotypes. These models consistently resulted in sustained anxiety-like behaviors and molecular alterations, including dysregulation of the stress axis, oxidative stress and neuroinflammation [8,19,48,49], and closely aligned with observations in mammalian models of PTSD, reinforcing the translational potential of zebrafish in neuropsychiatric research [1,2]. The study addressed its core research question by systematically analyzing how different PTSD paradigms in zebrafish influence behavioral, neurochemical, and molecular outcomes compared to unstressed controls, and identifying the most reliable stress induction protocols. Chronic paradigms—particularly medium-term CUS/UCS—consistently outperformed acute or social defeat models in producing persistent PTSD-like effects. Behavioral outcomes were reported in 93.9% of studies, with anxiety-like behaviors being the most prevalent phenotype. The NTT and LDT emerged as the most sensitive assays, especially when used in combination with social interaction metrics. Given the central role of social preference and shoaling behavior in zebrafish, the regulation of these behaviors by oxytocin receptor signaling, as demonstrated by Gemmer et al. (2022), highlights an important neuroendocrine axis that may influence susceptibility to social withdrawal under chronic stress conditions [50].

To further contextualize the behavioral phenotypes observed in zebrafish PTSD models, we compared them to well-characterized responses in rodent paradigms and clinical symptom domains [2]. Zebrafish exposed to chronic or trauma-like stress exhibit anxiety-like behaviors such as bottom-dwelling, thigmotaxis, reduced exploration, and increased freezing, which correspond closely to elevated anxiety and avoidance behaviors reported in rodent models such as the elevated plus maze, open field test, and contextual fear conditioning. Social withdrawal in zebrafish—typically assessed through shoaling disruption or diminished social preference—also mirrors reduced social interaction in socially defeated rodents and trauma-exposed human populations [51]. Notably, zebrafish also demonstrate cognitive impairments following trauma, including impaired fear memory and reduced avoidance learning, supporting their utility in modeling neurocognitive aspects of PTSD.

While zebrafish have traditionally been considered limited in their capacity to model trauma-related memory or re-experiencing symptoms, recent studies suggest otherwise. For instance, zebrafish exposed to a single life-threatening stressor display sustained context-dependent anxiety-like behavior and long-term behavioral alterations extending into adulthood, even in the absence of re-exposure [28,46,47]. These findings suggest the presence of persistent memory traces and stress-associated behavioral sensitization, which approximate certain dimensions of trauma recall and memory generalization. Therefore, while zebrafish cannot replicate complex cognitive features such as verbal intrusions or nightmares, they do demonstrate behavioral correlates of fear memory and persistent avoidance, reinforcing their relevance for modeling core PTSD symptom domains. A cross-species comparison of behavioral phenotypes is presented in Table 5.

Neurochemically, chronic stress induced frequent dysregulation in serotonin and dopamine systems, paralleling PTSD-related monoaminergic alterations in humans. Molecular changes included consistent upregulation of BDNF and IL-6 expression (1.8- and 2.3-fold increases, respectively), genes linked to neuroplasticity and inflammation [1,8]. Proteomic analyses further identified 18 differentially expressed proteins, with significant involvement of mitochondrial, oxidative, and glycolytic pathways, reflecting known mitochondrial dysfunction in stress-related disorders [18,31].

Cortisol dysregulation was observed in 56.5% of studies, making it the most commonly reported biomarker in zebrafish PTSD models [8,9]. However, these findings require cautious interpretation [49]. Chronic stress induced mean cortisol elevations of 42–58% versus controls, sustained for 7–21 days post-exposure [30], yet these responses appear to reflect generalized stress activation rather than PTSD-specific neuroendocrine dysfunction [19,52]. Clinical research similarly shows inconsistent associations between cortisol and PTSD. Some studies report elevated cortisol under acute stress [53], while meta-analyses show no significant differences in baseline cortisol between PTSD patients and trauma-exposed controls [54]. Furthermore, several studies found no correlation between hair cortisol concentration and PTSD severity [41,55], although others link HCC to core PTSD features [43]. In zebrafish, cortisol outcomes were strongly modulated by experimental design. Predator exposure increased cortisol levels by 72%, whereas social isolation led to a decrease, which normalized after brief re-socialization [56,57,58]. Cortisol levels typically peaked 21–40 min after stress and returned to baseline within 60 min in acute stress paradigms [59]. Significant sex differences were also reported, with females exhibiting 38% higher cortisol responses [9]. These findings emphasize that cortisol should not be used in isolation, but rather interpreted in conjunction with behavioral and molecular data to improve specificity. For example, combining cortisol elevation with social withdrawal correctly identified 81% of PTSD-like cases, while cortisol alone achieved only 56% specificity [54].

This review identifies several critical areas for advancing zebrafish-based PTSD research. First, real-time, non-terminal biomarker tracking is urgently needed. Current cortisol measurement techniques are terminal and limit longitudinal insights. Fluorescent glucocorticoid reporters and miniaturized biosensors could enable dynamic neuroendocrine profiling. Second, epigenetic regulation remains severely underexplored. Despite robust evidence linking methylation of nr3c1 and fkbp5 to PTSD in humans, none of the zebrafish studies systematically investigated epigenomic changes. Emerging technologies such as single-cell ATAC-seq in telencephalic neurons could uncover conserved regulatory mechanisms [60,61].

Third, environmental factors—such as housing density, water quality, and light cycles—are known to affect zebrafish stress reactivity but were poorly controlled across studies [1]. Recent evidence suggests that ambient light adaptation in zebrafish is modulated by key HPI-axis components, including *nr3c1* and *mc2r*, further highlighting the relevance of standardizing lighting conditions in behavioral stress assays [30]. Establishing standardized environmental conditions and using automated systems for population regulation could improve reproducibility. Finally, robust cross-species validation pipelines are needed to bridge the translational gap. A tiered approach—beginning with mechanistic testing in human iPSC-derived neurons, proceeding to rodent behavioral validation, and culminating in biomarker confirmation in clinical cohorts—would enhance external validity and align zebrafish findings with human PTSD biology [2].

In summary, this review systematically evaluated zebrafish PTSD models in terms of paradigm reliability, behavioral validity, neurochemical and molecular outcomes, and methodological consistency. The evidence supports medium-term CUS/UCS as the most effective and reproducible model. Zebrafish exhibit conserved behavioral and molecular stress responses and are a valuable platform for high-throughput screening of biomarkers and therapeutic agents. Future progress will depend on methodological standardization, integration of sex-specific analyses, expansion into epigenetic and longitudinal biomarker domains, and strategic cross-species validation. When used in this refined capacity, zebrafish can serve as a cornerstone for translational PTSD research and contribute meaningfully to the development of targeted interventions for trauma-related disorders.

## 5. Conclusions

This systematic review comprehensively evaluated 33 studies applying experimental PTSD paradigms in zebrafish, with particular focus on behavioral, neurochemical, and molecular outcomes. The evidence supports CUS/UCS protocols lasting 14–15 days as the most effective and reproducible models for inducing PTSD-like phenotypes. These paradigms consistently elicited anxiety-like behaviors, cortisol dysregulation, and changes in gene expression linked to inflammation and neuroplasticity, paralleling key dimensions of PTSD observed in mammals.

Importantly, the zebrafish models reviewed here do not attempt to fully replicate human PTSD, but rather to simulate specific endophenotypes such as hyperarousal, social withdrawal, and HPA-axis dysregulation. Within this framework, zebrafish represent a scalable, genetically tractable platform for identifying molecular markers and screening pharmacological interventions related to trauma-related disorders.

The review also highlighted significant methodological variability across studies, especially in statistical analysis approaches and experimental design. The inclusion of a comparative overview of statistical rigor and construct/predictive validity supports a strong rationale for increased standardization in future research. This includes the use of power analysis, effect size reporting, consistent behavioral metrics, and harmonized endpoints.

To advance the translational relevance of zebrafish PTSD models, we recommend prioritizing the following: (1) sex-stratified analyses to account for dimorphic stress responses; (2) longitudinal designs capturing delayed and persistent molecular changes; (3) integration of epigenetic, neuroimmune, and metabolic data; and (4) cross-species validation pipelines involving rodent models and human clinical samples. When utilized within this refined methodological framework, zebrafish can significantly accelerate the identification of reliable biomarkers and mechanistic pathways underpinning trauma-related psychopathology.

## 6. Limitations of the Study

This review identified several important limitations in the current state of zebrafish PTSD research, which should be considered when interpreting the results and planning future studies:

- Anatomical and Physiological Differences: Despite anatomical homologies, significant differences exist between zebrafish and human brain architecture that may limit the translational value of certain findings. The zebrafish telencephalon, while functionally analogous to the mammalian amygdala and hippocampus, exhibits distinct organizational patterns that could impact stress response mechanisms [1].
- Methodological Variability: Considerable heterogeneity exists in experimental protocols across studies, including stress induction methods, duration, and intensity; behavioral assessment paradigms; and analytical approaches. This variability compromises direct comparisons between studies and potentially affects reproducibility. Our analysis revealed that only a small proportion of studies explicitly reported power calculations, and nearly 30% exhibited inadequate control group quality. Standardization of methodologies is crucial for improving the reliability and translational value of zebrafish PTSD models.
- Limited Sex-Specific Analyses: One of the most critical gaps identified in this review is the insufficient exploration of sex differences in zebrafish PTSD responses. Few studies have compared male and female zebrafish, despite growing evidence that stress responses differ significantly between sexes in humans. This limitation is particularly significant given the well-documented sex differences in PTSD prevalence and symptomatology in clinical populations. Future research should incorporate sex-based analyses to improve the translational relevance of zebrafish PTSD models.
- Technical Limitations in Assessment: Current zebrafish models face technical constraints in monitoring long-term stress responses due to the inability to obtain sufficient blood samples without euthanizing the animal. This limitation hinders longitudinal studies of stress hormone dynamics and restricts the comprehensive assessment of neuroendocrine adaptations over time. Additionally, the relatively small size of zebrafish brains presents challenges for region-specific analyses of neurochemical and molecular changes.
- Cognitive and Emotional Complexity: Zebrafish lack the complex cognitive and emotional aspects of PTSD that humans experience, limiting their utility for modeling certain psychological symptoms [1]. While zebrafish exhibit anxiety-like behaviors and social withdrawal, they cannot replicate the intrusive thoughts, nightmares, or complex emotional processing characteristic of human PTSD. This fundamental limitation necessitates caution when extrapolating findings from zebrafish to human PTSD pathophysiology and treatment.
- Pharmacological Considerations: Although pharmacological interventions such as NAC and fluoxetine have shown promising results in zebrafish PTSD models [17], drug metabolism and pharmacokinetics differ substantially between fish and humans. The aquatic environment also introduces unique challenges for drug administration and dosing, potentially affecting the predictive validity of pharmacological studies. Furthermore, the effects of drug combinations and long-term treatments remain incompletely characterized in zebrafish PTSD models.

## Figures and Tables

**Table 1:** Comparative overview of zebrafish PTSD models, stressor types, primary outcomes, and endpoint timings.

Model Type	Duration	Stressors Used	Behavioral Outcomes	Neurochemical/Molecular Findings	Primary Outcomes	Endpoint Timing
Acute stress: osmotic + physical	4 min + 2 h recovery	Osmotic shock + netting	Reduced exploration, stress reactivity	↑ CRH, PAC1, otp	Behavioral responses, gene expression	2 h post-stress
Acute stress: combined (crowding/chasing/cold)	90 min	Crowding, chasing, cold shock	Freezing, bottom-dwelling, hyperactivity	Stress/inflammatory gene shifts	Anxiety-like behavior, gene expression	48 h post-stress
Acute stress: novel environment	Immediate (30–60 min)	Novel environment exposure	Group freezing, disrupted cohesion	↑ Cortisol	Group behavior, cortisol levels	30 min post-exposure
Innate stress (novel tank test)	10 min	Depth change, novelty	Bottom-dwelling, anxiety-like behavior	↑ HPI activation	Anxiety-like behavior	Immediately post-test
CUS/UCS (7 days)	7 days	Tank change, predator, temperature, restraint	Anxiety, oxidative stress, gene regulation	↑ Cortisol, ↓ GR, oxidative markers	Anxiety-like behavior, oxidative stress, gene regulation	24 h post-stress
CUS/UCS/PCS (10–15 days)	10–15 days	Predator, light, netting, chase	Anxiety, social changes, gene regulation	↑ CRF, BDNF, IL-6	Behavior, cortisol, gene expression, social interaction	24 h post last exposure
CUS/PUCS (3–5 weeks)	3–5 weeks	Unpredictable mixed physical/psychological stressors	Sustained anxiety, social avoidance	↑ cytokines, ↓ BDNF	Anxiety-like behavior, social withdrawal, cytokine shifts	Post 5-week exposure
PCUS (11 weeks)	11 weeks	Extended mixed unpredictable stressors	Persistent anxiety, neurotransmitter shift	Monoamine shifts	Persistent neurotransmitter shifts	Post 15-day exposure
Social defeat	6 days	Aggressive confrontation	Reduced social interaction, motivation	↑ Habenula activity, stress memory	Social preference, motivation	Day 7–8 post-defeat
Predator Threat (CAS)	Single event	Alarm substance	Avoidance, freezing	Nitrergic signaling	Anxiety-like behavior	Same day post-stimulus
Time-dependent sensitization	24 h	Shock context re-exposure	Sensitized stress reactivity	Altered nitrergic signaling	Stress sensitization	24 h post-exposure
Chemical stress (varied)	Varied	Dexamethasone, ethanol, crowding	Shoaling disruption, dark preference	Dopaminergic + neuroimmune signaling	Dark preference, shoaling disruption	Post-intervention behavioral testing
High-intensity trauma (THIT)	Not specified	Triple hit (visual, tactile, olfactory)	Contextual anxiety and sensitization	HPI-adrenaline interaction	Anxiety-like behavior	1–14 days post trauma

*Note: ↑ indicates an increase in hormone/indicator levels; ↓ indicates a decrease.*

**Table 2:** Summary of fish tanks and behavioral test timelines.

Behavioral Test Type	Tank Dimensions (Typical or Reported)	Common Timeline of Testing	Reported in Studies
Novel tank diving test (NTT)	28 × 15 × 7 cm; 24 × 15 × 10 cm	24 h to 14 days post-stress; 5–6 min trials	[7,17,41]
Open field test (OFT)	15 × 15 × 5 cm; 20 × 20 × 10 cm	Immediately to 7 days post-stress; 5 min trials	[33,35]
Light–dark preference test (LDT)	20 × 10 × 10 cm; 25 × 15 × 10 cm	Day 1 or 2 post-stress; 5–10 min	[19,36]
Alarm reaction test (AR)	10 × 20 × 20 cm; variable with predator/CAS exposure	Minutes to hours post-CAS/predator; 3–5 min	[30,37]
Shoaling/social interaction test	30 × 15 × 15 cm; 35 × 20 × 15 cm	1–3 days post-stress; ~10 min social approach testing	[9,39]
Conditioned place avoidance (CPA)	Two-chamber: each ~15 × 10 × 10 cm	Conditioning + testing phase on day 2–3 post-stress	[18]

**Table 3:** Neurochemical and molecular changes.

Model Type	Duration	Neurochemical/Molecular Findings	Representative Studies
Acute Stress	4–90 min	↑ CRH, otp, PAC1; immediate early gene activation	[20,33]
Short-term CUS/UCS (7 days)	7 days	↑ Cortisol, ↓ GR, ↑ CRF	[7,9]
Medium-term CUS/UCS (10–15 days)	10–15 days	↑ Cortisol, ↑ CRF, ↑ BDNF, ↑ IL-6, oxidative stress	[8,19]
Long-term CUS/PUCS (3–5 weeks)	3–5 weeks	↑ Cytokines (IL-1β, TNF-α), ↓ BDNF	[16,21]
Prolonged CUS (11 weeks)	11 weeks	Monoamine imbalance, chronic neurotransmitter shifts	[17]
Social defeat stress	6 days	↑ Habenula activity, stress memory markers	[24]
Early life stress	1–6 dpf	↓ POMC intensity, altered stress circuits	[38]
Chemical stress models	3–5 days	↓ Dopamine signaling, altered stress hormone response	[39]
Time-dependent sensitization	Acute + 24 h	Altered nitrergic signaling	[36]

*Note: ↑ indicates an increase in hormone/indicator levels; ↓ indicates a decrease.*

**Table 4:** Construct and predictive validity across zebrafish PTSD paradigms.

Model Type	Construct Validity	Predictive Validity	Notes
CUS/UCS (10–15 days)	High: Reproduces persistent anxiety, social withdrawal, HPI activation	Moderate to high: Responsive to fluoxetine, NAC	Most consistent model; parallels mammalian stress literature
Acute combined stress	Moderate: Induces transient anxiety, some HPI-axis activation	Moderate: Pharmacological response shown in select studies	Short-term; limited persistence of PTSD-like symptoms
High-intensity trauma (THIT)	Moderate to high: PTSD-like anxiety sustained for 14 days	Unknown/not tested	Strong reactivity but limited pharmacological validation
Social defeat stress	Variable: Social withdrawal observed; aggression context-sensitive	Low to moderate: No consistent pharmacological validation reported	High inter-study variability; needs protocol refinement
Prolonged UCS (PCUS/PUCS)	High: Persistent behavioral and cytokine changes	Moderate: Response to fluoxetine shown in one study	Potential for long-term modeling; requires replication
Developmental ELS/isolation	Moderate: Alters stress responsivity and social behavior	Low: Mixed drug effects	Useful for modeling susceptibility; not PTSD-specific

**Table 5:** Zebrafish vs. rat PTSD models.

Behavioral Domain	Observed in Zebrafish	Observed in Rats
Hyperarousal/startle response	Increased startle response (e.g., looming stimulus, CAS)	Exaggerated acoustic startle response, hypervigilance (SPS, footshock)
Avoidance behavior	Reduced top zone exploration, dark area preference (NTT, LDT)	Avoidance of trauma-associated context or cues (CPA)
Freezing/immobilization	Freezing, reduced mobility after shock/isolation	Freezing in conditioned fear paradigms
Social withdrawal	Reduced shoaling, impaired social approach after stress	Reduced social interaction post-stress (social defeat, SPS)
Cognitive impairment (learning, memory)	Impaired fear learning, reduced conditioned avoidance, disrupted retention	Impaired spatial learning (Morris water maze), contextual fear extinction
Contextual fear/traumatic memory recall	Persistent context-associated anxiety after single trauma exposure [46,47]	Persistent avoidance or fear reinstatement following trauma cue re-exposure
Circadian disruption/sleep disturbances	Impaired circadian rhythm (altered light/dark cycle or early-life stress)	Fragmented sleep, altered REM patterns (SPS, predator exposure)
Erratic/anxiety-like locomotion	Erratic swimming, thigmotaxis, reduced exploration	Reduced open field exploration, increased grooming
Aggression or irritability	Suppressed aggression (cold stress, confinement)	Model-dependent increase or suppression (social defeat, variable stress)

## Data Availability

No new experimental data were created.

## Abbreviations

The following abbreviations are used in this manuscript:

BDNF	Brain-derived neurotrophic factor
CUS	Chronic unpredictable stress
HCC	Hair cortisol concentration
HPA	Hypothalamic–pituitary–adrenal
HPI	Hypothalamic–pituitary–interrenal
IL-6	Interleukin 6
LDT	Light–dark test
NTT	Novel tank test
PCUS	Prolonged chronic unpredictable stress
PTSD	Post-traumatic stress disorder
PUCS	Prolonged unpredictable chronic stress
SYRCLE	Systematic Review Centre for Laboratory Animal Experimentation
UCS	Unpredictable chronic stress
